# Circadian rhythm genes and immune cell infiltration in myasthenia gravis: A comprehensive analysis

**DOI:** 10.1371/journal.pone.0327829

**Published:** 2025-07-07

**Authors:** Ke Wang, Peng Xu, Jing Lu, Xinchen Ji, Ying Zhang, Yibin Zhang, Dongxu Li, Dongmei Zhang, Tianye Lan, Jian Wang

**Affiliations:** 1 College of Traditional Chinese Medicine, Changchun University of Chinese Medicine, Changchun, China; 2 Department of Neurology, The Affiliated Hospital to Changchun University to Chinese Medicine, Changchun, China; 3 Research Center of Traditional Chinese Medicine, The Affiliated Hospital to Changchun University to Chinese Medicine, Changchun, China; 4 Scientific Research Office, The Affiliated Hospital to Changchun University to Chinese Medicine, Changchun, China; University of Mississippi School of Pharmacy, UNITED STATES OF AMERICA

## Abstract

The fluctuating weakness in myasthenia gravis (MG) is clinically described as the “morning improvement and evening worsening” pattern; MG is commonly associated with sleep disorders. However, there remains a paucity of research investigating the relationship between MG and circadian rhythms. This study seeks to identify pivotal circadian rhythm genes (CRGs) and characterize immune cell infiltration in MG, while exploring their potential roles in MG pathogenesis. MG data were obtained from the Gene Expression Omnibus (GEO) database. Initially, differentially expressed circadian rhythm genes between MG and control samples were identified through differential expression analysis. Subsequently, to elucidate the functional roles of differentially expressed CRGs, we conducted Gene Ontology (GO) and Kyoto Encyclopedia of Genes and Genomes (KEGG) pathway analyses. Finally, weighted gene co-expression network analysis (WGCNA) and least absolute shrinkage and selection operator (LASSO) regression were applied to identify the hub CRGs. The diagnostic utility of hub genes was evaluated using the receiver operating characteristic curve, and their protein expression levels in the serum of patients with MG were assessed utilizing enzyme-linked immunosorbent assay. Additionally, we examined the extent of immune cell infiltration in MG and explored its relationship with the identified hub genes. We analyzed the immune infiltration profile in MG and their correlation with the identified hub genes. The GO enrichment analysis revealed significant enrichment of differentially expressed genes in circadian rhythm-related biological processes. Our investigation identified two hub CRGs that exhibit high diagnostic specificity and sensitivity and are significantly upregulated in serum samples from MG patients. Furthermore, Immune cells were correlated with hub genes. Our findings suggest a potential circadian rhythm disorder in MG, which may offer novel biomarkers and therapeutic strategies for future research.

## Introduction

Myasthenia gravis (MG), classified as an organ-specific autoimmune disorder, predominantly disrupts neuromuscular junction (NMJ) function. Its clinical hallmark manifests as fluctuating skeletal muscle weakness and fatigue, clinically described as the “morning improvement and evening worsening” phenomenon [1–4]. Although the variability of this symptom is widely recognized, the mechanism remains unclear. MG exhibits an annual incidence rate of 10–29 cases per million person-years, with a corresponding prevalence rate of 100–350 cases per million individuals [5–7]. The pathogenesis of MG involves autoantibodies targeting neuromuscular junction proteins, including the acetylcholine receptor (AChR), muscle-specific kinase (MuSK), and low-density lipoprotein receptor-related protein 4 (LRP4), as well as thymic abnormalities and immune dysregulation influenced by genetic and environmental factors [4,8]. Despite advances in immunotherapy, individualization of treatment remains challenging and requires more in-depth mechanistic studies.

The circadian rhythm is a pervasive phenomenon observed across a diverse array of living organisms on Earth, encompassing Homo sapiens, fauna, and extending even to microbial kingdoms such as fungi and prokaryotic bacteria. The circadian rhythm is crucial for regulating various bodily functions, including sleep patterns, thermoregulation, appetite regulation, immune responses, and endocrine function. It is regulated by a intricate molecular circuitry of transcriptional-translational feedback loops, which coordinate multiple physiological mechanisms and behavioral output. The circadian rhythm exhibits an approximately 24-hour cycle physiological processes, ensuring the synchronization of internal biological processes with daily environmental cycles. The circadian rhythm is intricately linked to overall bodily health. Disruptions in the circadian rhythm can manifest at various levels, ranging from internal alterations in molecular, cellular, and tissue functions to imbalances among different tissues and physiological systems, as well as between behavioral patterns and environmental cycles [9]. The circadian rhythm serves as a modulator of numerous physiological processes and diseases. Dysregulation of circadian rhythms may trigger multisystem pathophysiological cascades, increasing susceptibility to various health conditions and significantly contributing to the onset and progression of illnesses. These include dysmetabolic syndromes such as obesity, diabetes, and hyperlipidemia [9,10]; neurodegenerative diseases such as Parkinson’s disease, Alzheimer’s disease, and frontotemporal dementia [11,12]; renal diseases including chronic nephritis and lupus nephritis [13]; cardiovascular diseases such as heart failure and myocardial infarction [14,15]; allergic diseases like asthma and atopic dermatitis [16,17]; and various cancers [18,19], all of which have been extensively studied. Recent emerging evidence reveals that the circadian clock machinery influences the onset and pathological escalation of autoimmune diseases through the regulation of immediate innate defenses and long-term adaptive immunity. [20,21]. Rheumatoid arthritis [22] and multiple sclerosis [23] have been associated with dysregulation of circadian rhythms, according to recent studies. Many patients with autoimmune diseases experience sleep disorders and fatigue. Multiple studies demonstrated that individuals with MG experience significant sleep disturbances [24–26], with sleep disorders being a primary manifestation of circadian rhythm disruptions. Additionally, MG frequently exhibit diurnal fluctuation of symptoms with morning improvement and evening exacerbation. Consequently, it is imperative to elucidate whether MG, as an autoimmune disease, is associated with circadian rhythm disturbances. Furthermore, elucidating the molecular basis of circadian rhythm disruption in MG will facilitate improvements in early diagnosis and the development of targeted therapeutic strategies.

**Fig 1:**
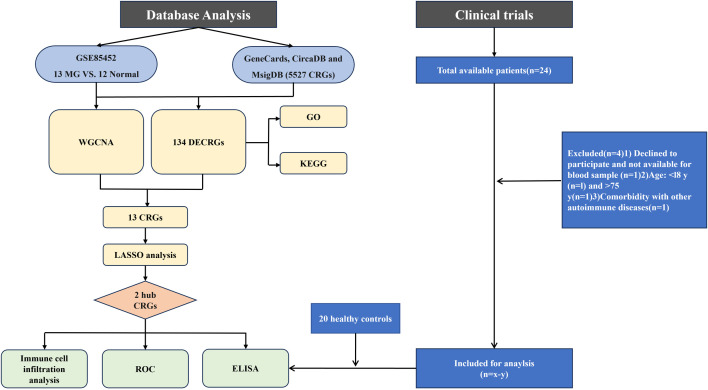
Flowchart outlining the analysis workflow. MG, myasthenia gravis; CRGs, circadian rhythm genes; WGCNA, weighted gene co-expression network analysis; DECRGs, differentially expressed circadian rhythm genes; GO, gene ontology; KEGG, kyoto encyclopedia of genes and genomes; LASSO, least absolute shrinkage and selection operator; ROC, receiver operating characteristic.

Bioinformatics analysis and enzyme-linked immunosorbent assay (ELISA) were applied to investigate circadian rhythm-related genes in MG. The study compared MG and control samples, identified hub genes, and performed functional enrichment and immune infiltration analyses to elucidate potential mechanisms. Subsequently, ELISA was applied to assess the transcript level of hub genes in serum samples from MG patients. The workflow of this study is presented in Fig 1. This study used bioinformatics methods to introduce the issue of MG circadian rhythm disorder and conducted a comprehensive discussion of the problem, in order to provide ideas for subsequent related research. Our research advances the identification of diagnostic biomarkers associated with circadian rhythm in MG patients, potentially elucidating the pathogenic mechanisms and identifying prospective therapeutic targets for circadian rhythm disorders in MG.

## Materials and methods

### Database analysis

#### Data collection and data processing.

This study utilized transcriptomic data from the Gene Expression Omnibus (GEO) database (https://www.ncbi.nlm.nih.gov/geo), with a focus on dataset GSE85452, which comprises genome-wide expression profiles of peripheral blood mononuclear cells (PBMCs) collected from 13 MG patients and 12 healthy controls. Additionally, we systematically curated a compendium of 5,527 circadian rhythm-associated genes through integration of three authoritative databases: GeneCards (https://www.genecards.org), CircaDB (http://circadb.org), and MsigDB (https://www.gsea-msigdb.org/gsea/msigdb). The circadian-related genes mainly included in this study encompass three categories: (1) core clock genes directly participating in the transcription-translation feedback loop (TTFL) of the molecular oscillator; (2) clock-modulating genes that indirectly affect rhythmicity by regulating the expression or function of core clock genes; and (3) clock-controlled output genes downstream of the core clock TTFL, whose rhythmic expression is driven by the oscillator to execute specific physiological functions [27–29]. This strategy was designed to comprehensively capture the chronobiological networks potentially affecting MG, rather than focusing exclusively on the core oscillator.

#### Differentially expressed CRGs screening.

The expression profiles of 9,630 genes were extracted from the GSE85452 dataset. The *limma* package was used to perform differential expression analysis, specifically employing the *lmFit()* function to construct linear models and the *eBayes()* function for empirical Bayes variance shrinkage. Statistical significance was defined by |log2FC| > 0.3 and *p* < 0.05. DECRGs between the MG and normal groups were identified using a Venn diagram. Subsequently, the *pheatmap* and *ggplot2* packages were employed to generate heatmaps and volcano plots for the visualization of DECRGs.

#### Function enrichment analysis.

The biological roles of DECRGs were analyzed using Gene Ontology (GO)/ Kyoto Encyclopedia of Genes and Genomes (KEGG) enrichment analysis via the *clusterProfiler* package, with a significance threshold of *p* < 0.05 and FDR < 0.05.

#### Weighted gene correlation network analysis (WGCNA).

To construct a gene co-expression network for MG, we employed the WGCNA package. Data preprocessing included quality checks (*goodSamplesGenes*) and outlier detection (*hclust*). An optimal soft threshold (R² ≥ 0.9) was selected using pickSoftThreshold to enforce scale-free topology. Pairwise correlations were used to build the network, with modules identified via hierarchical clustering and dynamic tree cutting (minModuleSize = 50). Modules were merged (mergeCutHeight = 0.25) if eigengene correlations > 0.75. Module significance (|r| > 0.3; *p* < 0.05, FDR < 0.05) and gene selection criteria (MM > 0.8, GS > 0.5) ensured biological relevance. Network refinement involved merging adjacent modules, followed by visualizations (labeledHeatmap, verboseScatterplot) to identify key modules, inter-module interactions, and MG-related core genes.

#### Identification and diagnostic efficiency of hub CRGs.

To identify MG-associated hub CRGs, the candidate hub genes were first intersected with DECRGs. Least Absolute Shrinkage and Selection Operator (LASSO) regression (*glmnet* package) was then applied to the overlapping genes, with cross-validation used to determine the optimal *λ*. The resulting hub CRGs were validated through differential expression analysis, visualized as a violin plot (*ggviolin*), and further evaluated for diagnostic potential using ROC curve analysis (*pROC* package).

#### Immune infiltration analysis.

Immune cell infiltration in MG was evaluated using CIBERSORT analysis on PBMCs derived from the GSE85452 dataset, comprising 13 MG patients and 12 healthy controls. The algorithm employed the LM22 reference matrix, which contains 22 immune cell types, for deconvolution analysis, performed with 1000 random permutations to assess reproducibility. The data consisted of log2-transformed and standardized FPKM values, which were used to filter out low-expression genes. Stacked bar charts were used to visualize the estimated relative abundances of 22 immune cell types across samples. The intergroup differences were further visualized through boxplots. The intergroup differences were further visualized through boxplots (Fig 5b). Following a normality test, we selected the Pearson method for correlation analysis and generated a heatmap to illustrate the relationships between hub circadian rhythm genes and immune cells.

**Fig 2:**
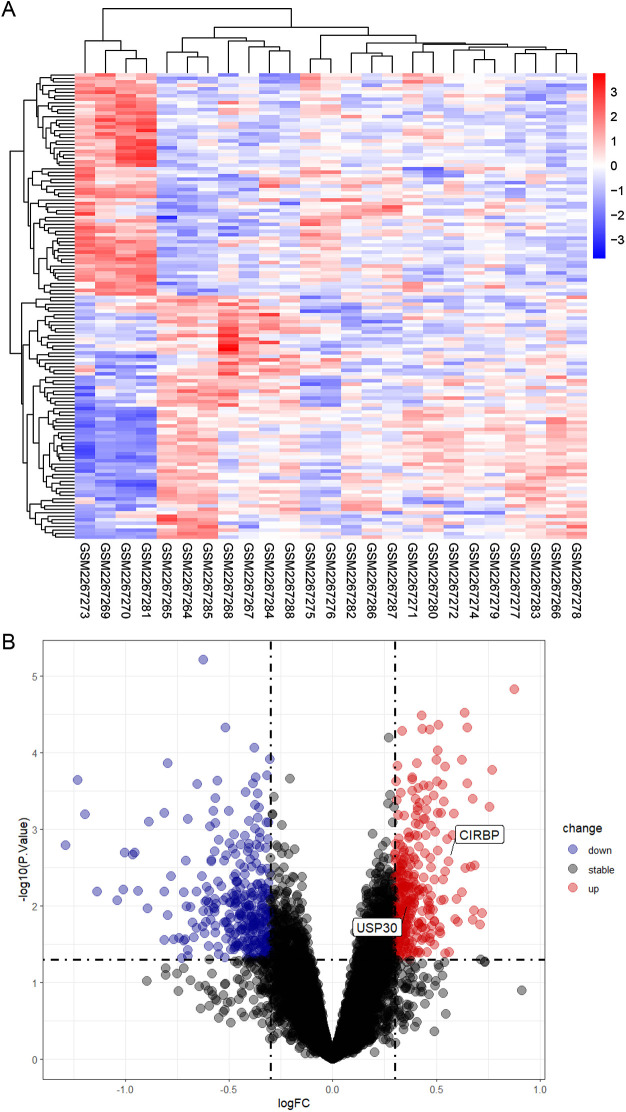
Identification of DECRGs between MG and normal samples. (a) The heatmap displays the gene expression levels of each sample; (b) Volcanic map shows DECRGs between two groups. Normal group: GSM2267264-68,8-80, 83-85,88; MG group: GSM2267269-77,81,82,86,87.

**Fig 3:**
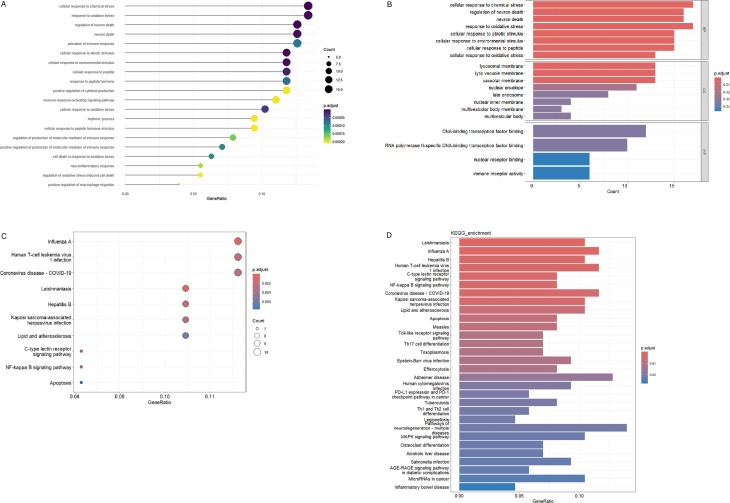
Enrichment analysis of DECRGs between MG and normal samples. (a) Lollipop plots of GO analysis results; (b) Bubble graph of GO analysis results; (c) Bar plot of KEGG pathway analysis results; (d) Bubble graph of KEGG pathway analysis results. BP, biological process; CC, cellular component, MF, Molecular function.

**Fig 4:**
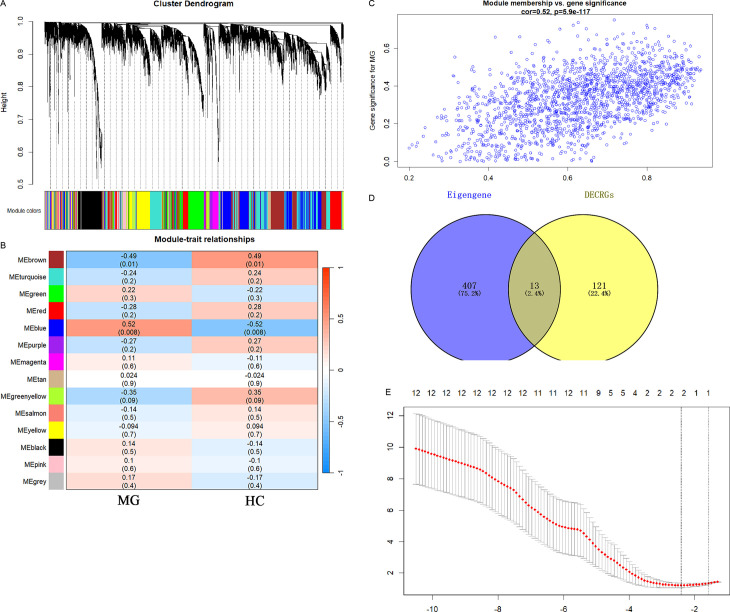
Screening of hub CRGs. (a) The cluster dendrogram of the genes. (b) Heatmap of the module-trait relationships. (c) Gene distribution within the blue module. (d) The intersection of the eigengene of the blue module and DECRGs; (e) LASSO regression analysis.

### Clinical validation

The study cohort comprised 20 patients diagnosed with MG who sought treatment at the Department of Neurology of the Affiliated Hospital of Changchun University of Chinese Medicine between March 2024 and July 2024. twenty healthy controls (HCs) were enrolled at the medical examination center. Peripheral blood samples were collected from both study cohorts, with serum subsequently isolated through standardized centrifugation (3000 rpm, 10 min, 4°C) for subsequent ELISA detection and quantitative analysis. This exploratory study aims to identify CRGs associated with differential protein expression in MG patients relative to healthy controls. Blood collection was standardized under fasting conditions within 8:30 a.m. ± 50 minutes to control for circadian variations. This research protocol received approval from the Ethics Committee of the Affiliated Hospital of Changchun University of Chinese Medicine. Written informed consent was acquired from all study participants. No minors were involved in this study.

#### Enzyme linked immunosorbent assay (ELISA).

Serum concentrations of human cold-induced RNA-binding protein (CIRBP) and ubiquitin-specific protease 30 (USP30) were quantitatively assessed using commercial ELISA kits (MEIMIAN Biotechnology, China) following the manufacturer’s protocols. The absorbance was determined with a multifunctional assay reader (BioTek Instruments, Inc., USA) at a wavelength of 450 nm. GraphPad Prism 10 was employed for nonparametric statistical testing and ROC curve analysis. Statistical significance thresholds were established at *p* < 0.05.

## Results

### Identification of differentially expressed genes

This study employed the *limma* package’s *lmFit()* function to construct linear models and applied the *eBayes()* function for empirical Bayes variance shrinkage, conducting differential expression analysis on 3,448 CRGs in the GSE85452 dataset. By setting significance thresholds at *p* < 0.05 and |log2FC| > 0.3. We identified 134 CRGs demonstrating significant differential expression between the MG group and normal controls. The heatmap visually displays clustering relationships among samples or genes, while clearly demonstrating differential expression patterns across multiple samples and genes (Fig 2a). The volcano plot provides a clearer representation of differentially expressed genes, revealing 64 upregulated and 70 downregulated genes (Fig 2b).

### Functional enrichment analysis of CRGs

Functional annotation of MG-associated CRGs through systematic GO enrichment analysis revealed significant enrichment across the tripartite GO classification: biological processes (BP), cellular components (CC), and molecular functions (MF). Specifically, in the context of biological processes, DECRGs were predominantly enriched in neuronal death, response to oxidative stress, positive regulation of immune response mediators, and circadian rhythms (Fig 3a,b; S2 Table). KEGG pathway analysis revealed significant enrichment of DECRGs in NF-κB signaling, C-type lectin receptor signaling, Toll-like receptor signaling, and T-helper cell differentiation pathways (Th1/Th2/Th17) (Fig 3c,d; S3 Table).

### WGCNA and identification of hub CRGs in MG

We identified 14 co-expression modules through WGCNA, each represented by distinct colors. The dendrogram delineates hierarchical clustering of co-expressed genes into distinct color-coded modules (Fig 4a). Module-trait correlation analysis demonstrated significant associations, with the blue module positively correlating with MG status (r = 0.52, *p* = 0.008) and the brown module showing inverse correlation (r = −0.49, *p* = 0.01) (Fig 4b,c). The blue module was identified as the pivotal gene cluster based on its maximal statistical significance. Intersection analysis between 134 DECRGs and 420 blue module hub candidates yielded 13 MG-associated CRGs (Fig 4d; S4 Table). LASSO regression modeling ultimately prioritized two core regulators: CIRBP (OR = 3.75) and USP30 (OR = 1.94) (Fig 4e).

### Immune infiltration analysis

**Fig 5:**
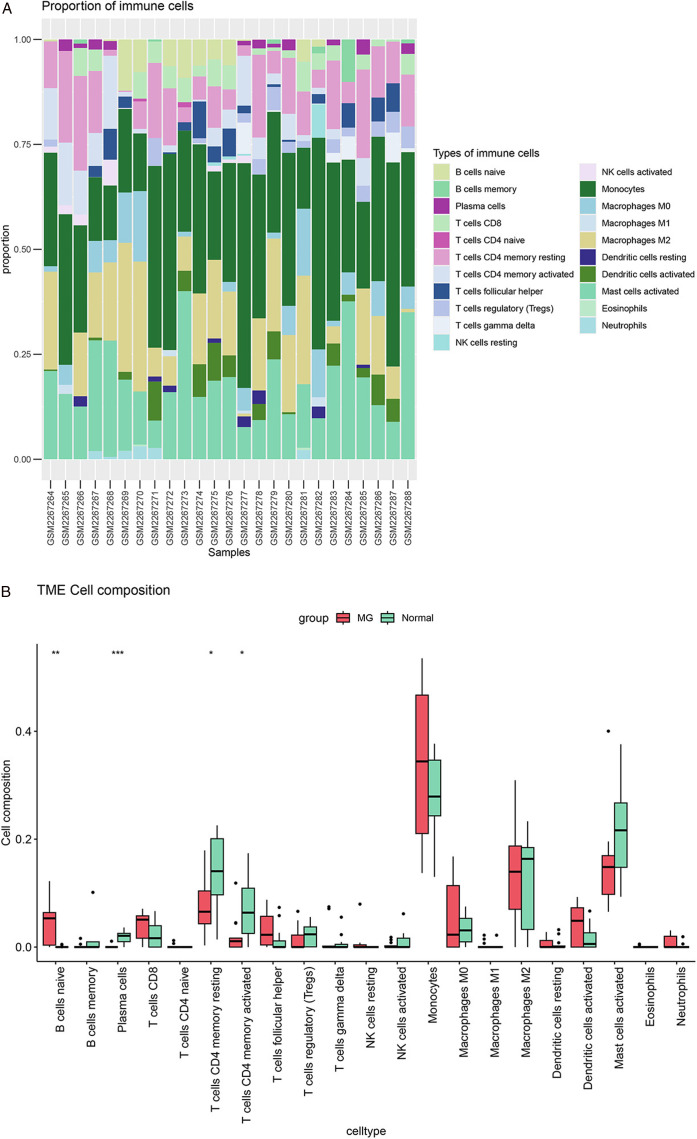
Immune cell infiltration. (a) The proportion of 22 immune cells in MG samples and normal samples; (b) The box plot shows the difference in immune cell infiltration levels between the MG group and normal samples. Normal group: GSM2267264-68,8-80, 83-85,88; MG group: GSM2267269-77,81,82,86,87.

Characterization of the MG immune microenvironment was achieved through computational deconvolution analysis using the CIBERSORT algorithm, which systematically quantified infiltration landscapes of 22 immune cell subtypes. Comparative profiling revealed significant disparities in lymphocyte subset proportions between MG patients and healthy controls (Fig 5a; S5 Table).Computational deconvolution revealed elevated naive B cell infiltration coupled with paradoxical depletion of antibody-secreting plasma cells and memory CD4^+^ T cell subsets (resting/activated) in MG patients versus controls (Fig 5b), implying multifactorial impairment of adaptive immunity, potentially involving defective B cell maturation and T cell memory formation.

### Correlation analysis of hub CRGs and infiltrating immune cells

**Fig 6:**
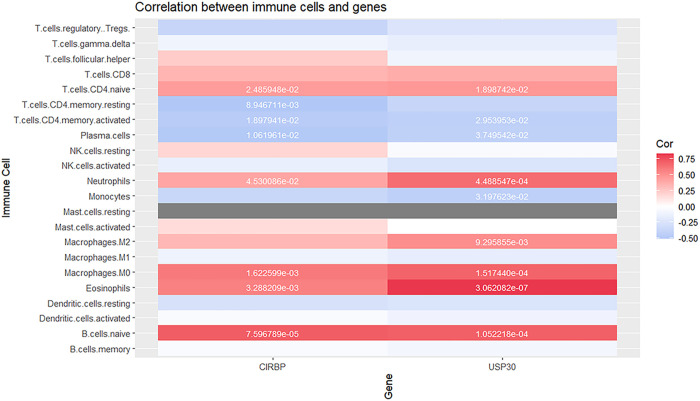
Correlation analysis of hub CRGs and infiltrating immune cells. Heatmap of the correlation between 22 immune cells and two hub genes. The red module represents a positive correlation and the blue module represents a negative correlation. The darker the color, the greater the correlation.

Immunomodulatory network analysis revealed distinct correlation patterns between hub CRGs (CIRBP/USP30) and immune infiltration profiles in MG patients: correlation analysis demonstrated both CRGs exhibited positive associations with naïve CD4^+^ T cells, naïve B cells, neutrophils, M0 macrophages, and eosinophils, while inversely correlating with activated memory CD4^+^ T cells and plasma cells. Notably, CIRBP specifically showed negative correlation with resting memory CD4^+^ T cells, whereas USP30 uniquely displayed positive association with M2 macrophages and negative correlation with monocytes (Fig 6). These results suggest circadian-modulated immune dysregulation, where CRGs temporally coordinate myeloid-lymphoid interactions in MG pathogenesis.

### Diagnostic value of hub CRGs

**Fig 7:**
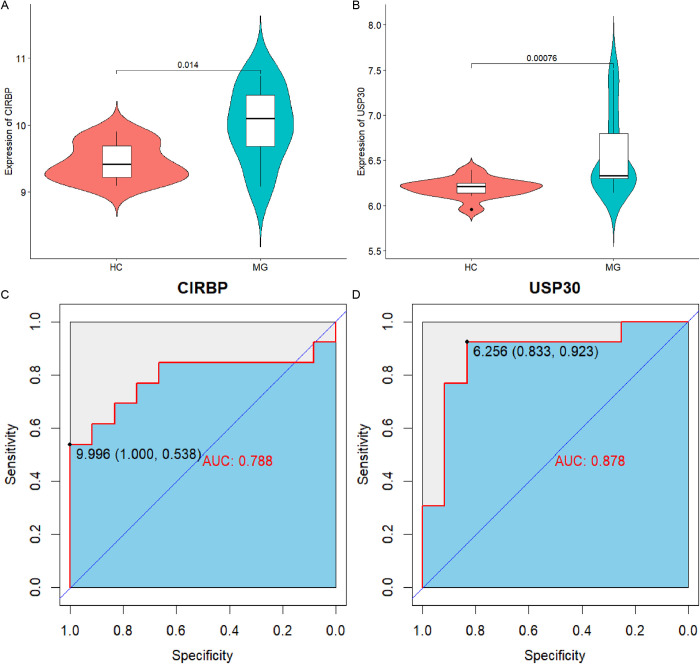
Expression levels and ROC curves of hub CRGs. (a-b) Differential expression of hub CRGs in MG and healthy samples; (c-d) The area under the ROC curve shows the diagnostic efficacy of two hub genes. AUC, area under the curve; HC, healthy control; MG, myasthenia gravis.

The differential expression analysis demonstrated that CIRBP and USP30 exhibited significantly elevated expression levels in the MG compared with healthy controls, as illustrated by the violin plots (Fig 7a,b). Subsequent evaluation of their diagnostic potential through receiver operating characteristic (ROC) curve quantification yielded area under curve (AUC) values of 0.79 for CIRBP and 0.88 for USP30 (Fig 7c–d). These results suggest strong specificity, minimal false positives, and reliable accuracy in distinguishing MG patients from controls.

### Protein concentration levels of CRGs in MG patients

**Fig 8:**
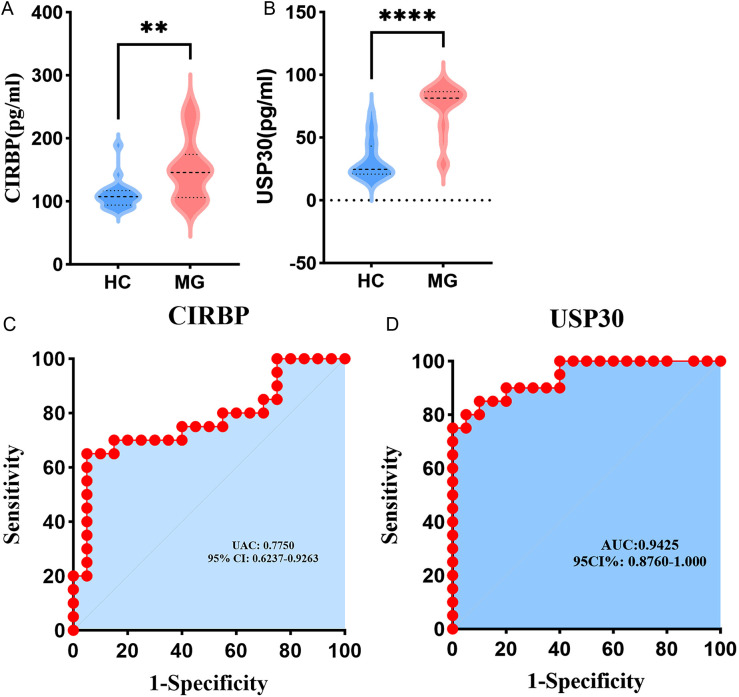
The Hub CRGs is highly expressed in the serum of MG patients. (a-b) Comparative analysis of CIRBP and USP30 serum concentrations between MG patients and healthy controls; (c-d) ROC curve of hub CRGs for MG diagnostic efficacy. ** p < 0.01, ****p < 0.0001. AUC, area under the curve; HC, healthy control; MG, myasthenia gravis.

To investigate the clinical relevance of identified hub genes (CIRBP/USP30) in MG pathogenesis, we conducted ELISA-based quantification of serum protein levels. Demographic parameters showed no intergroup differences, confirming group comparability (Table 1). Statistical analysis demonstrated significantly elevated protein levels of CIRBP and USP30 in MG patients versus controls (Fig 8a,b). Additionally, the AUC values for CIRBP and USP30 were 0.7750 and 0.9425, respectively (Fig 8c,d). These results corroborate our bioinformatics analysis at the protein level, underscoring the potential clinical utility of CIRBP and USP30 as biomarkers for MG.

**Table 1:** Clinical characteristics of MG patients and healthy controls.

Variables	Healthy control group (n = 20)	Myasthenia gravis group (n = 20)	P
Female, n (%)	11 (55%)	11 (55%)	>0.9999
Age (years)	50 (44.50–55.75)	52.50 (45.25–58.50)	0.4482
CIRBP (pg/ml)	107.30 (94.26–117.30)	145.90 (106.00–174.40)	0.0024
USP30(pg/ml)	24.79 (20.90–43.31)	81.42 (66.19–86.53)	<0.0001

## Discussion

MG, a chronic immune-mediated condition targeting neuromuscular junctions, manifests through pathogenic autoantibody interference with synaptic transmission mechanisms, frequently accompanied by sleep disturbances [30] and circadian rhythm abnormalities. In this study, we identified hub genes associated with circadian rhythm in MG using bioinformatics approaches, revealing that CIRBP and USP30 are upregulated in monocytes. Furthermore, we investigated the interplay between these genetic elements and distinct subsets of immune cells, and conducted a preliminary investigation into the biological roles of MG-related CRGs, elucidating potential pathways and molecular functions. In addition, we validated the hub gene results through ELISA on the serum of MG patients, discovering elevated protein expression levels of CIRBP and USP30. The dysregulation of circadian clock genes adversely impacts on the immune system, and this study may elucidate the potential factors in the development of MG and potential therapeutic targets.

CIRBP is a key regulator in mammalian cells responding to cold stress [31]. A study published in *Science* in 2012 demonstrated that CIRBP is essential for the robust expression of circadian genes [32]. CIRBP enhances the amplitude of the circadian oscillator by facilitating cytoplasmic accumulation of mRNA that encodes the Clock Circadian Regulator (CLOCK). In fibroblasts where CIRBP expression is knocked down, there is a marked reduction in the accumulation of CLOCK [32]. Furthermore, the presence of CIRBP was found to orchestrate the transcriptional dynamics of three quantitative clock genes, which subsequently modulating the cerebral cortex’s response to sleep deprivation [33]. These findings collectively position CIRBP as a circadian auxiliary regulatory factor, bridging environmental stressors (e.g., sleep deprivation) to the robustness of circadian oscillations, with potential implications for diseases like MG where both circadian disruption and immune dysregulation coexist. As the first evidence linking CIRBP to MG, our data demonstrate a significant upregulation of CIRBP in MG patient. Nevertheless, the functional contribution to CIRBP in MG disease progression remains unclear, and its potential mechanisms (e.g., autoimmune activation, circadian rhythm disruption) require further exploration. While no existing literature directly elucidates the pathological role of CIRBP in MG, its potential relevance can be inferred from its established biological functions and immunomodulatory mechanisms. Notably, CIRBP not only serves as a regulator of circadian rhythm genes but also participates in innate immune responses through multiple pathways. Prior studies have demonstrated that CIRBP directly interacts with the LPS receptor TLR4, triggering the secretion of inflammatory signaling molecules including TNF-α and IL-6, which establishes its role as an endogenous regulator of inflammatory cascades in physiological contexts [34]. Further experimental evidence reveals that approximately 77% of inflammation-related genes exhibit overexpression in CIRBP-knockout cells, accompanied by hyperresponsiveness to TNF stimulation [35], which corroborates the negative regulatory role of CIRBP in immune homeostasis from a reverse genetics perspective. Although CIRBP remains unexplored in MG pathogenesis, emerging evidence from autoimmune research has established significant associations between elevated CIRBP expression and clinical severity in rheumatoid arthritis [36] and systemic sclerosis [37]. Our research extends these findings to MG, demonstrating that elevated CIRBP levels represent a ubiquitous phenomenon in autoimmune disorders, suggesting a shared mechanism of immune dysregulation. This cross-disease validation strengthens the hypothesis that CIRBP dysregulation represents a conserved pathogenic mechanism in autoimmune disorders, potentially serving as a novel therapeutic target for MG management. Although no direct studies have investigated the relationship between CIRBP and MG, we propose a potential link based on CIRBP’s established role in immune regulation and the recognition of circadian clock genes as contributing factors in autoimmune disorders [38]. Our findings provide preliminary evidence suggesting CIRBP’s potential involvement in MG pathogenesis, though the precise molecular mechanisms require further elucidation.

USP30 can modulate ubiquitin tagging on OMM-anchored substrates, which in turn regulates mitophagy. USP30, as a core negative regulator of mitochondrial autophagy, inhibits mitochondrial autophagy by antagonizing the ubiquitination of Parkin [39–41], thereby affecting the dual pathogenic mechanisms of circadian rhythm and MG. Based on current research evidence, we postulate that USP30 may indirectly regulate circadian rhythms through its functional roles, e.g., inhibiting mitophagy and its ubiquitination. First, mitochondrial autophagy maintains circadian rhythm stability by selectively degrading NR1D1 and regulating BMAL1 expression [42]. Upregulation of USP30 hinders mitochondrial autophagy, leading to the accumulation of NR1D1 and inhibition of BMAL1 expression, triggering a cascade reaction that disrupts the biological clock.Furthermore, sleep deprivation amplifies pathological mitochondrial accumulation in neuronal populations, while mitochondrial signaling serves as a central conduit mediating the bidirectional relationship between sleep architecture and cerebral energy homeostasis [43,44]. These findings collectively demonstrate that sleep disorders and mitochondrial dysfunction form a self-reinforcing pathogenic feedback loop, underscoring the intricate interplay between mitochondrial integrity and circadian regulation. Parkin directly regulates the transcriptional activity of circadian clock genes (e.g., per, tim, and clk) [45]. Meanwhile, USP30 antagonizes Parkin’s ubiquitination function [40]. This suggests USP30 may indirectly participate in circadian rhythm regulation by inhibiting Parkin, though its specific contribution requires verification.

Emerging evidence indicates that the mitochondrial antioxidant enzyme levels are influenced by the circadian timing system through transcriptional regulation, thereby orchestrating redox homeostasis [46]. Intriguingly, USP30—a mitochondria-anchored deubiquitinating enzyme—exerts ROS-scavenging activity through modulating mitochondrial protein quality control systems [47]. Given the bidirectional crosstalk between ROS and circadian rhythms (i.e., circadian-driven ROS production and ROS-mediated clock gene regulation) [48], we propose a novel regulatory axis wherein USP30 fine-tunes mitochondrial ROS flux, potentially serving as a chronobiological modulator that couples mitochondrial dynamics to circadian transcriptional networks.

The ubiquitin-proteasome system critically orchestrates circadian regulation through distinct enzymatic mechanisms. USP2, a circadian deubiquitinating enzyme (DUB) transcriptionally activated by the CLOCK/BMAL1 complex [49], modulates BMAL1 protein abundance and proteolytic turnover [50]. Similarly, USP7 stabilizes core clock proteins Cry1 and Cry2 via deubiquitinating, thereby fine-tuning circadian rhythm oscillations [51]. Although USP30 primarily localizes to mitochondria, other DUBs such as USP2 and USP7 directly regulate core clock proteins. This mechanistic evidence implies that USP30 may indirectly modulate circadian rhythms via analogous DUB-related regulatory pathways.

Mitophagy dysfunction exhibits strong pathological correlations with MG progression. Studies demonstrate that impaired mitophagy significantly reduces muscle cell regenerative capacity, thereby exacerbating neuromuscular transmission deficits critical for NMJ maintenance [52,53]. Li et al. suggest that mitochondrial dysfunction in skeletal muscle may represent a potential mechanism contributing to MG pathogenesis [54].Our study identifies significant upregulation of USP30 in MG patients, consistent with previous findings. Collectively, USP30’s dual regulatory roles—mitochondrial quality control and circadian modulation—may position it as a central hub linking MG pathology with circadian dysfunction. However, this study cannot fully exclude the possibility that USP30 impacts MG pathogenesis through non-circadian pathways (e.g., isolated mitochondrial dysfunction). Future experiments should validate its circadian specificity. Consequently, CIRBP and USP30 may be identified as clock-regulated genes implicated in circadian rhythm disturbances associated with MG. Nevertheless, the precise relationship and specific mechanisms linking MG with CIRBP and USP30 remain inadequately understood and warrant further investigation.

Autoimmune diseases and circadian rhythms exhibit a bidirectional regulatory relationship, wherein dysregulation of either component can precipitate a deleterious feedback loop [55,56]. Circadian rhythm disorders are usually manifested as sleep disorders. Patients with MG exhibit a high prevalence of sleep disturbances, with significantly elevated sleep disorder scores compared to the general population [26,57]. Notably, active-phase MG patients show a greater incidence of pathological Pittsburgh Sleep Quality Index (PSQI) scores compared to those in clinical remission [58], while well-treated stable MG patients maintain improved nighttime sleep quality [59].Importantly, sleep deficiency further aggravates fatigue in MG, establishing a detrimental feedback loop [60]. A cohort study highlights that chronic sleep disruption may increase susceptibility to autoimmune diseases via dysregulated inflammation and compromised immune tolerance, underscoring the therapeutic potential of sleep interventions for long-term risk reduction [61]. Collectively, longitudinal analyses reveal sleep-wake cycle disruptions a positive correlate with accelerated symptom exacerbation in MG patients., implying that sleep impairment is more likely to emerge as MG progresses and may serve as a potential dynamic monitoring indicator for MG activity.

Current research indicates that circadian dysregulation and disruption are implicated in a broad spectrum of pathological conditions. The appropriate application of circadian rhythm science may facilitate an improvement in the therapeutic index. The appropriate application of circadian rhythm science has the potential to enhance drug efficacy, thereby offering additional benefits in disease treatment and management. Increasingly, studies have corroborated that pharmacokinetics can be modulated to produce time-specific toxicity and efficacy via transcriptional-translational feedback loops, which are governed by the biological clock as the central regulator of these rhythms [62,63]. Optimizing or correcting the biological clock, particularly in the context of circadian rhythm-related diseases, has the potential to transform a detrimental cycle into a beneficial one, thereby ameliorating the condition. Zhou et al. demonstrated through animal experiments that the efficacy of theophylline in treating colitis significantly varied with the timing of administration [64]. Furthermore, a crossover clinical trial investigating the timing of rivaroxaban administration revealed that nighttime dosing resulted in superior therapeutic efficacy and safety compared to morning dosing [65]. Numerous clinical drugs have demonstrated time-dependent effects, exemplified by the recognized efficacy of administering antihypertensive medications at bedtime [66–68], Chronotherapy has also been applied to various diseases, including glioma [69], depression [70], multiple sclerosis [71], rheumatoid arthritis [72], and asthma [73]. Beyond the timing of medication, elements including light exposure, nutritional interventions, exercise, and lifestyle modifications have shown potential in rectifying circadian clock desynchrony [74–76]. Circadian rhythm medicine, an emerging discipline, optimizes personalized treatment through integration of biological clock mechanisms. Chronotherapeutic strategies based on circadian principles—including pharmacological dose management synchronized to patients’ circadian rhythm profiles [77] and drug delivery systems aligned with endogenous physiological rhythms [78]—demonstrate reduced adverse effects and enhanced therapeutic efficacy. A comprehensive understanding of circadian biology remains pivotal for advancing individualized and precision medical practices [9].

MG is a cell-dependent autoimmune disorder characterized by the involvement of T lymphocytes, B lymphocytes, dendritic cells (DCs), and natural killer (NK) cells. Consequently, this study performed an analysis of immune cell infiltration utilizing data from the GEO database (GSE85452) to elucidate the relationship between various immune cell types, their functional states, as well as its circadian hub genes. In this study, immune infiltration analysis of PBMCs from MG patients, conducted using deconvolution algorithms with the LM22 reference matrix, revealed significant correlations between hub CRGs and multiple immune cell populations, including T lymphocytes, B lymphocytes, macrophages, eosinophils, and neutrophils. Notably, plasma cells directly contribute to the pathogenesis of MG through autoantibody secretion. However, our study observed downregulation of plasma cell abundance, which may be attributed to both the inherent limitations of the CIBERSORT algorithm in detecting low-abundance cell types [79] and the restricted sample size. Future investigations of immune infiltration in MG should employ more precise and sensitive computational algorithms combined with statistically robust sample sizes to enhance analytical reliability.

Methodological boundaries of this investigation warrant explicit articulation. Firstly, the potential exclusion of significant genes due to the limited sample size in public databases. Secondly, the constraints associated with the available resources in these preclude validation against external datasets. To address these limitations, this study further corroborated the findings by analyzing peripheral blood samples from clinical patients to enhance the accuracy of the results. Future research should leverage advanced technologies to facilitate validation across both the proteomic and mRNA dimensions in clinical and basic experimental contexts. Additionally, single time-point serum measurements are insufficient to characterize dynamic proteomic changes in MG, and may additionally miss hub genes exhibiting regulated circadian oscillations. Future investigations should implement longitudinal time-series sampling (e.g., collecting specimens at 4–6-hour intervals over 24–48 hours) coupled with circadian rhythm detection algorithms (e.g., *JTK_Cycle* and *MetaCycle*.). This approach will enable precise identification of candidate genes and oscillatory parameters (phase/amplitude) driving circadian rhythm perturbations in both preclinical models and human MG cohorts. Furthermore, longitudinal profiling can distinguish pathological circadian dysregulation from stochastic transcriptional variation, thereby providing mechanistic insights into diurnal symptom variability.

## Conclusions

In summary, this study employed WGCNA and LASSO analysis to identify two hub genes associated with the circadian rhythm in MG. The clinical diagnostic efficacy of these genes was validated through ROC curves and ELISA. Additionally, these genes exhibited a significant association with immune cells, suggesting that the disruption of immune cell balance, excessive secretion of inflammatory cytokines, and abnormal expression of circadian rhythm genes are interrelated processes that collectively influence the pathogenesis and progression of MG. Our findings elucidated novel pathophysiological insights into MG comorbid with circadian rhythm disorders, identified candidate biomarkers. These insights present a fresh perspective on MG regulation of circadian rhythm function and suggest potential drug-targeted strategies for MG treatment. However, these preliminary findings warrant multicenter prospective studies to confirm clinical relevance while elucidating the biological pathways involved. Future research could also prioritize optimizing the interplay between diseases and circadian rhythm disorders through refined medication timing and lifestyle interventions.

## Supporting information

S1 Table DECRGs between the MG and control groups. (XLSX)

S2 Table Gene Ontology enrichment analysis result. (XLSX)

S3 Table Kyoto Encyclopedia of Genes and Genomes pathway analysis result. (XLSX)

S4 Table Blue module candidate hub genes. (XLSX)

S5 Table Proportion of 22 kinds of immune cells in two groups of samples. (XLSX)
